# Immunization against GnRF in adult cattle: a prospective field study

**DOI:** 10.1186/s12917-017-1129-x

**Published:** 2017-07-01

**Authors:** Gaby Hirsbrunner, Sarah Rigert, Fredi Janett, Jürg Hüsler, Philipp Schnydrig, Ester Lopez, Sue Amatayakul-Chantler, Adrian Steiner

**Affiliations:** 1 Clinic for Ruminants, Vetsuisse Faculty, University of Berne, Bremgartenstrasse 109a, CH-3012 Berne, Switzerland; 2 Tierarztpraxis Arche, CH-3952 Susten, Switzerland; 3 Department of Farm Animals, Vetsuisse-Faculty, University of Zurich, Winterthurerstrasse 260, CH-8057 Zurich, Switzerland; 4 Institute of Mathematical Statistics and Actuarial Science, Sidlerstrasse 5, CH-3012 Berne, Switzerland; 5 Institut for Veterinary Bacteriology, Länggassstrasse 122, CH-3012 Berne, Switzerland; 6 Zoetis Research and Manufacturing, 45 Poplar Road, Parkville, VIC A-3052 Australia

**Keywords:** Cattle, Anti-GnRF, GnRH, Immunization, Estrus, Antibody titer, Eringer breed

## Abstract

**Background:**

Suppression of cyclic activity in cattle is often desired in alpine farming and for feedlot cattle, not intended for breeding. A cattle specific anti-GnRF vaccine (Bopriva™) is registered for use in heifers and bulls in different countries. In adult cows vaccinated with Bopriva™, the median period until recurrence of class III follicles was 78 days from the day of the 2nd vaccination and reversibility could be proven, as out of 11 experimental cows 10 cows became pregnant at first, and one cow at second insemination. In the present study, 76 healthy, cyclic Eringer heifers and cows were vaccinated twice with Bopriva™ 3-7 weeks apart, to prevent estrus during alpine pasturing. Blood samples were taken for progesterone and GnRF antibody titer analysis on the day of inclusion (7–9 d before the first vaccination) and at the first vaccination. At the same time, gynaecological examinations were performed. When estrus occurred in the course of the alpine pasturing season, a gynaecological examination was done including analysis of a blood sample (progesterone, anti-GnRF antibody titer). Cows were followed for fertility out to 26 months post second vaccination.

**Results:**

Median duration of estrus suppression was 191 days after the second vaccination (when the 2 vaccinations were given 28–35 days apart). From *n* = 13 cows showing signs of estrus on the alpine pasture, *n* = 7 could not be confirmed in estrus (serum progesterone value >2 ng/ml, no class III follicles seen using ultrasonography). Median duration between second vaccination and next calving was 496 days (25%/75% quartiles: 478/532 days).

**Conclusion:**

Bopriva™ induced a reliable and reversible suppression of estrus for more than 3 months in over 90% of the cows.

**Electronic supplementary material:**

The online version of this article (doi:10.1186/s12917-017-1129-x) contains supplementary material, which is available to authorized users.

## Background

Estrus suppression is desired by cattle owners for feedlot animals not intended for breeding and for animals in seasonal breeding management (e.g. summer pasturing in the Alps). Young heifers and older beef cows are often unintentionally pregnant at slaughter (Federal Food Safety and Veterinary Office: Projekt Schlachtung von trächtigen Rindern – Prävalenz und Gründe der Schlachtung). Immunization against GnRF has been used to reduce sexual behavior and to prevent the development of secondary sexual characteristics in males [1–8], and for suppression of estrous cycles [9] and prevention of unwanted pregnancies in females [10, 11]. GnRF antibody titers, ovarian function, progesterone concentration and uterine weight differed significantly between immunized and non-immunized heifers [9]. In adult cows vaccinated with Bopriva™ (Zoetis Australia Limited, 38–42 Wharf Road, West Ryde, NSW 2114, Australia), the median period until recurrence of class III follicles (> 9 mm in diameter and less than 2 cm; [12]) was 78 days from the day of the 2nd vaccination [13]. It was also shown that the absence of class III follicles was the best clinical indicator of anestrus.

Estrous behavior of the cows, though, was inhibited for 30 weeks (180 days after second vaccination). Reversibility could be proven, as 10 out of 11 cows fell pregnant soon after first, and 1 cow after the second insemination [13]. In pastures with commingling of cows from different farms of origin, non-pregnant cows may cause agitation and accidents in the herd because of recurring estrous behavior. This is especially accentuated in the Swiss Eringer breed, raised for fighting and selected for dominance abilities [14]. Estrus suppression during pasture commingling is highly valued, but currently, with the exception of intravaginal progesterone devices, no pharmaceuticals are approved in Switzerland for suppression of cycling activity in food-producing animals. Immunization against GnRF is a likely animal-friendly alternative. Therefore, the aim of this prospective study was to evaluate the duration and efficiency of an active immunization against GnRF using Bopriva™ for estrus suppression in free range adult Eringer cattle during mountain pasturing in the Swiss Alps. Their fertility variables in the subsequent breeding season were described. We hypothesized that the vaccination induced estrus suppression would last longer than 80 days after the 2nd vaccination and that it would be reversible.

## Methods

### Animals, inclusion and exclusion criteria

From April to May 2014, *n* = 76 heifers and cows of the Eringer breed (confirmed non-pregnant) spending the summer free ranging on the Alps with daily supervision were included in the study. Animals suffering from purulent vaginal discharge, inseminated less than 30 days before and with a history of recurring ovarian cysts were excluded from the study. The history of the cows and the structure of the farms were investigated by a questionnaire for the owners. Calving dates and number of inseminations in the preceding and subsequent season were additionally sourced from the Swiss animal movement data base (www.agate.ch) and the Eringer breeding association.

### Gynaecological examination

The cows were examined twice (at inclusion and first vaccination) within 7–9 days including a vaginoscopy, a rectal palpation of the genital tract and an ultrasonographical examination. Uterine symmetry and tone, cervical opening and properties, and amount of vaginal discharge were recorded [15]. Using ultrasonography (Magic 500, Eickemeyer, Eickemeyer Medizintechnik, Appenzell, Schweiz), the number of follicles was recorded and assigned to 1 of 3 categories, according to the classification of Moreira et al. [16]: class I follicles (≤5 mm in diameter), class II follicles (6–9 mm in diameter), and class III follicles (>9 mm and <2 cm in diameter) [12]. The presence of a corpus luteum (CL) was also noted.

### Blood sampling, GnRF antibody titer and progesterone

In all animals, blood was sampled at inclusion, at first and second vaccination and at the time of the first heat after estrus suppression. The blood samples were collected by venipuncture from the coccygeal vein into heparinized tubes, centrifuged (4000 x g, 10 min), and plasma was then stored at −18 °C for later analysis.

GnRF antibody titers were determined by dissociation enhanced lanthanide fluorescence immunoassay (DELFIA) (PerkinElmer Pty Ltd., Glen Waverly, Australia), and results were expressed as relative light units (RLU). Briefly, 384-well streptavidin coated plates (PerkinElmer Pty Ltd., Glen Waverly, Australia) were coated for 1 h at room temperature with 1 μg/mL biotinylated modified GnRH peptide in DELFIA buffer (50 mM Tris-HCl, 0.9% NaCl, 0.05% Tween 20, 20 μM EDTA, 0.2% ovalbumin). Plates were washed and then incubated for 1 h at RT with 50 μL aliquots of cattle samples serially diluted to 1/800 in DELFIA buffer. Unbound plasma and antibodies were removed by washing, and bound antibody was detected by incubating plates for a further 1 h with 50 μL of europium labeled protein G (PerkinElmer Pty Ltd., Glen Waverly, Australia). After washing off excess europium labeled protein G, DELFIA Enhancement Solution (PerkinElmer Pty Ltd., Glen Waverly, Australia) was added to all wells to dissociate bound lanthanide from the antigen forming a highly fluorescent chelate. After 10 min, the intensity of fluorescence was measured by excitation at 340 nm and emission readings at 615 nm using a time resolved fluorometer (Envision 2102 Multireader, Perkin Elmer). Pooled non-vaccinated cattle samples served as a negative control. Unknown samples were compared to serial dilutions of a standard positive reference immune cattle sample. Calculation of a standard curve and reading of unknown samples were performed using the WorkOut 2.5 software (Dazdaq Solutions Ltd., East Sussex, England). Intra- and inter-assay CV were 6.7% and 8.5%, respectively. Serology titers showed a 120 fold range from 3850 RLU titers units for negative samples to 495,000 titers units for immune samples, with 11,500 RLU being taken as the lower cut-off differentiating positive from negative samples. Lowest detection level of GnRF antibody titer was 3850 units [13].

Progesterone concentration was measured using radioimmunoassay (Immunotech, Beckman Coulter, Sinsheim, Germany). The intra-assay coefficient of variation (CV)% was 5.3% and the inter-assay CV% 7.7% determined by using a bovine pool serum, measured 30 times within a test routine and between 25 assay runs. The analytical sensitivity was 0.05 ng/mL and the measurement range was 0.05–50 ng/mL. The assay was performed according to the manufacturer’s instructions.

### Treatment; GnRF vaccine

The treatment consisted of two vaccinations with the anti-GnRF vaccine Bopriva™ (Bopriva, Zoetis Australia Limited, 38–42 Wharf Road, West Ryde, NSW 2114, Australia) 4 weeks apart whenever applicable. Bopriva™ contains an analogue of GnRF linked to a carrier protein accompanied by a synthetic aqueous adjuvant (400 μg GnRF-protein-conjugate per mL) and is registered with claims to suppress testosterone blood levels in post-pubertal bulls as well as to suppress estrous behavior in post-pubertal heifers. The dosage used for both the initial and the booster vaccination was 400 μg GnRF-protein-conjugate (1 mL Bopriva™). All injections were administered subcutaneously on the right side of the neck.

All animal experimentation was performed with permission and in accordance to Swiss law (https://www.blv.admin.ch/blv/en/home/tiere/tierversuche.html; Nr. G54/3616).

### Clinical evaluation, side effects

Behaviour and food intake was carefully observed by the owners. If activity and / or food intake was judged to be reduced for more than 25%, a veterinarian was consulted. The injection site was palpated for signs of heat, swelling and sensitivity daily for 1 week. Estrous behavior observation was conducted daily during alpine pasturing by the responsible herdsmen.

### Follow-up

Owners were instructed to immediately report if animals showed estrous behavior. When estrous behavior was observed, a gynaecological examination was performed and another blood sample was taken for analysis of GnRF-antibody titer and serum progesterone concentration. In the cows’ first heat after the alpine season, blood samples were planned to be taken and examined.

All owners completed a second questionnaire concerning the first heat of the cows, number of inseminations until next pregnancy and reasons for leaving the farm. Data of subsequent calving was taken from the Swiss animal transportation internet portal (www.agate.ch).

### Statistical analysis

The continuous variables are described as mean, sd, median and quartiles, the categorical with absolute and relative frequencies. The primary endpoint “period from 2^nd^ vaccination to estrus” was analyzed with the nonparametric Kaplan-Meier survival function, since some of the cows were slaughtered before coming in heat which is considered as censored time periods. The different periods of the different groups were compared with the log rank test. Several explanatory factors were investigated with the Cox regression. A *p*-value <0.05 indicated a significant result.

## Results

### Animals, inclusion and exclusion criteria

Ten cows and heifers were excluded because of a missing second vaccination, culling due to other reasons than fertility problems during the alpine pasturing season. Seventy-six animals (13 heifers and 63 cows) were included. Before the alpine pasturing season 90% of the owners took care of their animals themselves, whereas 10% of the owners used a communal cattle shed. All owners preferred seasonal calving from September to December. Concerning the use of the cows, 58.3% of the owners indicated to milk them for a mean of 180 days, 37.5% used them as beef (suckling) cows for a mean of 120 days, and 4.2% of the owners neither milked them nor let the calf suckle. Mean herd size was 10 cows and 4 heifers, 96% of these animals spent the summer on the Alps.

### Cows’ histories

Median age of the cows and heifers was 5.3 yr. (range: 1.8–10.9 yr). First calving age of the cows was 3.1 yr. (median; range 1.8–4.8 yr). Preceding intercalving period was 445.9 days (mean; range: 311–779 days). Conception index for the preceding pregnancy was 1.5 (median), conception rate at first insemination was 50% and at second insemination was 51.7%. Signs of heat were considered “good” in 67.6%, “moderate” in 26.5% and “not observed” in 5.9% according to the owner’s judgment.

Culling rate due to fertility problems was 9.4%.

### Gynaecological examination at inclusion / first vaccination

On the day of inclusion, 10 animals suffered from an urovagina. At the time of inclusion and time of first vaccination 67 and 70 animals, respectively had a small uterus that was retractable onto the floor of the pelvis. None of the animals had proliferations of the most caudal cervical ring. A functional CL was present in 46 and 47 of cattle at the time of inclusion and the time of the first vaccination, respectively. Median number of class I-II-III follicles was 2–1-0 at inclusion and 2–1-1 at first vaccination (Additional file 1).

### GnRF vaccine; side effects

The 2 vaccinations were performed 20–49 days apart (median 32 days). None of the owners identified any serious adverse side effects in their cows. Most of the cows treated with Bopriva™ showed a slight swelling at the injection site, varying in severity, and moderate local pain for 2–9 days after vaccinations.

### GnRF antibody titer and progesterone

The GnRF antibody titer at the inclusion of the study and on the day of the first vaccination was below the detection limit (3800 units). At the second vaccination, the titers ranged between 3850 and 236,998 units (median: 17,870 units) in 62 cows, while in 12 cows, the titer was still below the detection limit (2 results missing). Median titers in heifers at second vaccination and at first signs of heat were higher compared to median values in cows (Table 1). Median antibody titer of animals seen with signs of heat was 3850 (range 3850–16,623).

**Table 1:** Anti-GnRF antibody titer (DELFIA) in heifers and pluriparous cows at first and second vaccination and at first signs of estrus on the Alp and after the alpine season

	heifers	pluriparous cows
First vaccination		
Median	3850	3850
25% quartile	3.850	3850
75% quartile	3850	3850
Second vaccination		
Median	28,919	15,520
25% quartile	19,501	10,939
75% quartile	41,899	22,494
Estrus signs on the Alp pasture (*n* = 1 heifer, *n* = 12 pluriparous cows)		
Median	3850	3850
25% quartile		3850
75% quartile		10,231
First signs of estrus after the alpine season		
Median	17,772	3850
25% quartile	13,885	3850
75% quartile	35,420	10,775

Serum progesterone values ranged from 0.3 to 40 ng/ml (median: 4.7 ng/ml) at first examination. At first vaccination, the serum progesterone levels ranged from 0.3–40 ng/ml (median: 6 ng/ml), with 15 cows ≤2 ng/ml. At the second vaccination, the serum progesterone levels ranged from 0.2–32.1 ng/ml (median: 2.8 ng/ml) with 33 cows ≤2 ng/ml.

### Vaccination induced anestrus

**Fig. 1:**
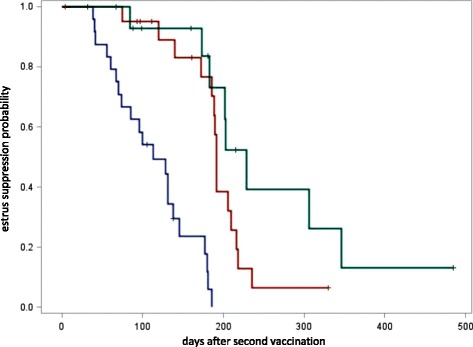
Kaplan Meyer graph representing the percentage of cattle within group 1–3, versus the duration of the period between second vaccination and estrus. The median times are 113, 191 and 229 days in the three groups. Group 1 (*blue*) = < 28 days, group 2 (*brown*) = 28–35 days, and group 3 (*green*) = > 35 days between the vaccinations. † mean censored data

One heifer and twelve cows showed signs of estrus while on pasture and 30 animals were detected after return to the farm of origin (alpine season 100–150 days depending on climate). Median duration of estrus suppression after the second vaccination was 113 days (<28 days between the 2 vaccinations), 191 days (28–35 days between the 2 vaccinations) and 229 days (>35 days between the 2 vaccinations). The cox regression was started with the period “2nd vaccination to heat” in days and the explanatory factors “cow”, “serum progesterone value at 1st and 2^nd^ vaccination” and the categorized variable “period between the 2 vaccinations”. The latter was grouped in group 1 (< 28 days), group 2 (28–35 days), and group 3 (> 35 days). The backward selection revealed only group 1 as a significant factor with *p* < 0.0001. Figure 1 shows the percentages of days between the second vaccination and heat in the three groups. Plasma progesterone at the time of the first and second vaccination did not affect duration of estrus suppression.

### Follow-up

Follow-up data were included until 26 months after the second vaccination. Out of the 76 cows, 35 cows were culled within a median of 134 days after the second vaccination. From these 35 cows, 23 were culled without being inseminated (planned to be culled after alpine pasturing), 7 were culled for fertility reasons and 5 for reasons other than fertility problems. Median duration between second vaccination and subsequent calving in 37 cows was 496 d. Calving data of four cows was not available, but they were pregnant in the 2015/2016 season.

## Discussion

Our hypothesis was confirmed: the cows’ estrus was suppressed for more than 80 d. The alpine pasturing season lasts for a minimum of 100 d, depending on the weather. Therefore, the length of cycle suppression for cows vaccinated with Bopriva™ was satisfactory, and depended on the length of the period between the 2 vaccinations (medians ranged from 113 to 229 days). To our knowledge, this variability in response in females has not been described yet, whereas in bulls it is known, that the duration of testosterone suppression is dependent on the type of adjuvant used, and the number of and the interval between the vaccinations (3–4 weeks versus 6–8 weeks) [17]. GnRF antibody titers at second vaccination and at first signs of heat were increased in heifers as compared to pluriparous cows. As only 17% of the animals included were heifers, a statistical difference could not be determined between the 2 groups. Only twelve cows and one heifer were observed with signs of estrus during alpine pasturing. However, seven of these animals had serum progesterone values >2 ng/ml, no class III follicles when examined on the alpine pasture, but median antibody titers were below the detection limit. In mares vaccinated against GnRF, estrous behavior occurred in anovulatory mares, as only small amounts of estradiol are needed for induction of estrous signs [18]. The characteristics of the old breed “Eringer” are known to be dominant and hot-tempered [14] and might be intensified in the alpine pasturing systems. In older bulls “learnt behaviors” may contribute to sexual behaviors that persist following vaccination with Bopriva™ (personal communication Sue Amatayakul-Chantler). It is possible that the behavioral signs of estrus in cattle may not always be related to findings on the ovaries and serum progesterone concentrations.

The GnRF antibody titer increases 2–3 weeks after the first vaccination and peaks 2 weeks after the second vaccination. Due to alpine pasturing logistics, it was not feasible to measure the anti GnRF antibody titer during the alpine season, except for those cows observed in heat in which serum samples could be taken on the Alps. Median duration between second vaccination and subsequent calving was 496 days. This period consists of 280 days pregnancy and 150 days of alpine pasturing and a voluntary waiting period of 50–70 days, as alpine pasturing and cow fight rules demand a seasonal calving, with 80% of the calvings occuring between October and December [19]. The calving month and seasonal calving were the most important reasons for a prolonged calving to conception interval in this breed [19]. Therefore, the evaluation of classic fertility variables is difficult in the Eringer cows and vaccinated with Bopriva™, as their preceding calving was between October 2012 and March 2014. Owners prefer to let the cows open instead of allowing them to calve during the wrong season of the year.

Unfortunately, 29% (23/76) of the cows were culled within 6 months after the alpine season e.g. before the end of the subsequent breeding season. For the owners of those cows, it was important that the estrous cycle of the cows be suppressed during the alpine season, so cows could be slaughtered in the fall or winter, when meat prices are higher. In the remaining cows, the culling rate due to fertility problems was 9.2% similar to 9.4% from the preceding season. Our results support those from a previous study that Bopriva™ does not negatively impact bovine fertility [13].

## Conclusion

We can highly recommend anti-GnRF vaccination (Bopriva™) for reversible suppression of estrus in Eringer cattle spending the summer on alpine pasture. For a period of 28–35 days between the two vaccinations anestrus is lasting up to 216 days with a median of 191 days. If a longer period of cycle suppression is desired, the period between the two vaccinations should be >35 days. Seventy percent of the cows calved in the subsequent calving seasons.

## Additional file

Additional file 1: Data of all cows included. Gynaecological examinations, dates of vaccinations, titers (anti-GnRF and progesterone at inclusion, at first vaccination, at 2nd vaccination, at first heat). (XLSX 51 kb)

## Abbreviations

CL: Corpus luteum
DELFIA: Dissociation enhanced lanthanide fluorescence immunoassay
GnRF: Gonadotropin Releasing Factor
GnRH: Gonadotropin Releasing Hormone
RLU: Relative light units
